# Influence of Ramped Compression on the Dielectric Behavior of the High-Voltage Epoxy Composites

**DOI:** 10.3390/polym13183150

**Published:** 2021-09-17

**Authors:** Muhammad Bilal Iqbal, Abraiz Khattak, Asghar Ali, M. Hassan Raza, Nasim Ullah, Ahmad Aziz Alahmadi, Adam Khan

**Affiliations:** 1 U.S.-Pakistan Center for Advanced Studies in Energy, High Voltage Laboratory, National University of Sciences and Technology (NUST), Sector H-12, Islamabad 44000, Pakistan; bilaliqbalmgt@gmail.com (M.B.I.); mhassanraza423@gmail.com (M.H.R.); 2 U.S.-Pakistan Center for Advanced Studies in Energy, Department of Energy System Engineering, National University of Sciences and Technology (NUST), Sector H-12, Islamabad 44000, Pakistan; asghar@uspcase.nust.edu.pk; 3 Department of Electrical Engineering, College of Engineering, Taif University KSA, P.O. Box 11099, Taif 21944, Saudi Arabia; nasimullah@tu.edu.sa (N.U.); aziz@tu.edu.sa (A.A.A.); 4 Department of Electronics Engineering, University of Engineering and Technology (UET) Peshawar (Abbottabad Campus), Abbottabad 22010, Pakistan; adamkhan@uetpeshawar.edu.pk

**Keywords:** epoxy, high voltage, dielectric properties, composite, insulation

## Abstract

The emergence of micro and nano-based inorganic oxide fillers with optimal filler-loadings further enhances the required insulation characteristics of neat epoxy. During manufacturing and service application, insulators and dielectrics face mechanical stresses which may alter their basic characteristics. Keeping this in mind, the facts’ influence of mechanical stresses and fillers on dielectric properties of polymeric insulators of two epoxy/silica composites were fabricated and thoroughly analyzed for dielectric characteristics under ramped mechanical compressions relative to the unfilled sample. Before compression, epoxy nanocomposites exhibited responses having an average dielectric constant of 7.68 with an average dissipation factor of 0.18. After each compression, dielectric properties of all samples were analyzed. The dissipation factor and the dielectric constant trends of each sample are plotted against a suitable frequency range. It was observed that after the successive compressions up to 25 MPa, the dielectric properties of epoxy micro-silica composites were highly affected, having an average final dielectric constant of 9.65 times that of the uncompressed sample and a dissipation factor of 2.2 times that of the uncompressed sample, and these were recorded.

## 1. Introduction

Polymeric insulators and dielectrics have been extensively utilized in electrical insulation and dielectric applications due to their superior properties over the conventional ceramic insulators, such as light in weight, flexibility in nature, ease of installation, low maintenance, low cost, higher tensile strength, and higher dielectric strength [1,2,3]. The dielectric behavior of polymeric dielectrics is of utmost importance for usage in energy storage applications [4]. Mechanical stresses significantly influence the dielectric properties of polymeric dielectrics [5,6]. Ramy et al. studied the effect of mechanical stresses on polymeric-insulating materials and concluded that electric field strength decreased significantly due to mechanical stresses. Shen et al. [7] studied the impact of hydrostatic compression on nano carbon-filled epoxy composites and deduced a decrease in electrical resistance due to decrement in the tunneling gap. Khattak et al. [8] studied the dielectric behavior of epoxy silica micro and nanocomposites at 15 MPa pressure. It was concluded that compression increased the dielectric constant and dissipation factor of all the composites. Shen et al. [9] conducted a study on dielectric properties of SnO_2_ under high pressure and found that the dielectric constant and loss tangent decreased with increasing frequency. Authors of [10] studied the effects of mechanical stresses on polymeric materials and concluded that all the critical phenomena, including a decrease of dielectric strength, electrical tree growth, accumulated damage, space charge accumulation, etc., in insulating polymer materials are significantly influenced by internal (residual) and external mechanical stresses. In another study, it was concluded that mechanical stresses alter the dielectric properties of epoxy due to the change of tetragonal orthorhombic structures of epoxy into cubic structures [11]. Epoxy is known for its remarkable dielectric properties and mechanical resilience [12,13]. However, pristine epoxy expresses degradation in dielectric properties due to mechanical stresses. To mitigate the effect of mechanical compression on epoxy, its composites with inorganic fillers are recommended. Inorganic fillers not only increase the mechanical strength but also improve the dielectric properties of epoxy [14,15]. Various inorganic oxide filler-based composites, for example, zinc oxide, titania, and alumina, are used to improve the dielectric characteristics of the neat epoxy [16,17]. This prompts the replacements of neat epoxy by its composites in electrical insulation and as dielectrics. So far, less study has been undertaken on dielectric properties of epoxy composites and their correlation to the compression. Single compression effects on the dielectric behavior of epoxy and its composite are not enough to understand the influence of compression on dielectric properties of epoxy. This is of utmost importance and will give detailed insights into the function of compressions with dielectric properties of neat epoxy, nano, and microcomposites.

Keeping in mind the above motivation, the purpose of this study is to explore the dielectric response of epoxy–silica nano and microcomposites, as well as their impact on multiple increasing successive compressions.

### Preparation of Nanocomposite

Bisphenol-A (DGEBA) (Eposchon^®^) resin diglycidyl ether, cycloaliphatic amine (EPH 555^®^) hardener, and 189 ± 5 g (equivalent to 86 g/eq of amine hydrogen) of epoxy were utilized. Justus Kimia Raya, Indonesia, provided both the resin and the hardener. Degussa, USA, provided Nanosilica (AEROSIL^®^ 200) with an average particle size of 12 nm with 200 m^2^/g of surface area. Micro-silica with an average particle size of 5 µm was used and it was provided by NewReach chemicals, Wuhan, China.

**Figure 1:**
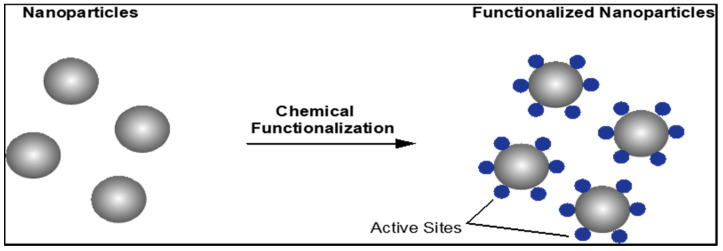
Schematic of the functionalization of nanoparticles.

The surface functionalization of silica was achieved with silane, which was provided by (DOW CORNING ^®^ Z-6040, Midland, TX, USA), whiles the solvent ethanol was obtained from Sigma-Aldrich^®^ from the United States. Surface functionalization was performed to achieve better filler-polymer interaction. The schematic of the functionalization process is shown in Figure 1. For preventing clinging, silica was poured and scattered into the ethanol. The mixture was placed for 1.5 h in an ultrasonic bath to obtain optimum dispersion. Using a high-shear mixer with a rotating speed of 3600 revolutions per minute, the epoxy resin was added in the mixture. The solution was then returned to the ultrasonic bath for another 30 min. Then, hardener was added into the mixture and it was rotated in the shear mixer at 7200 revolutions per minute for 30 min. Then, the solution was vacuum-sealed at temperatures over the boiling point of ethanol to allow for all the ethanol to be evaporated. The slurry was kept in vacuum at 27 mmHg for degassing and debubbling for 15 min. In the following stage, the mixture was moved to castings and kept for 24 h at 25 °C. In the last stage, samples were cured for approximately 4 h. The prepared samples have an 80-mm diameter and a 3-mm thickness. Table 1 shows a list of the prepared samples with their codes.

## 2. Measurements and Methods

### 2.1. Dielectric Properties Measurement

The dielectric constant (ĸ) can be defined as:

$$Dielectric\;Constant=\frac{Permittivity\;of\;Dielectric\;material}{Permittivity\;of\;free\;space}$$

(1) $$ĸ\;=\;\frac{\varepsilon_{m}}{\varepsilon_{0}}\;$$

The capacitance of a capacitor may be determined using the relation:

(2) $$\;C\;=\;\;\frac{A\varepsilon }{d}$$

where, *A* = area of the capacitor plate;

*ε* = permittivity; and

*d* = thickness of the dielectric.

As a result, the dielectric constant (ĸ) can be calculated by dividing the capacitance of the capacitor with a dielectric material (*C_m_*) by the value of the capacitance (*C*_0_) with air medium, expressed as:

(3) $$ĸ=\;\frac{C_{m}}{C_{0}}$$

To measure dielectric properties, the LCR meter 7600 Plus with a dielectric cell LD-3 from IET Labs, USA, was utilized.

### 2.2. Compression Setup and Conditions

**Figure 2:**
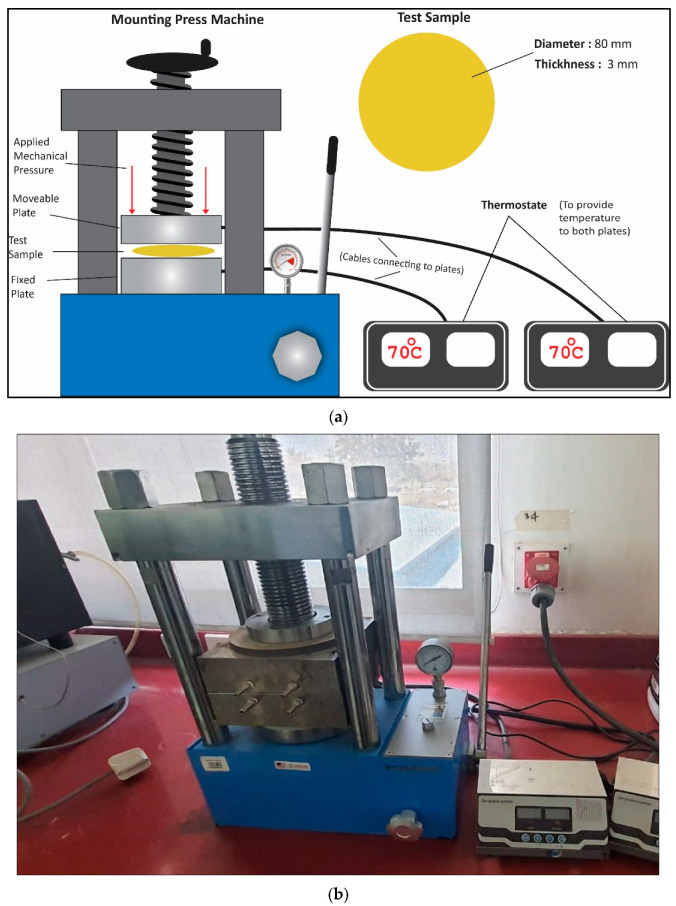
Schematic diagram of the compression setup. (**a**,**b**) Photo of the compression setup and its pictorial view.

Mechanical compression setup was comprised of a CY-600D mounting press, made in China, connected with thermostats for controlled heating purposes. Epoxy composite samples were first heated at 700C and then compressed. The compression experiment was carried out with five constant increasing interval mechanical pressure steps, i.e., 5 MPa, 10 MPa, 15 MPa, 20 MPa, and 25 MPa. The schematic representation of the compression setup and its pictorial view are shown in Figure 2. 

## 3. Results and Discussion

### 3.1. Results at Low Frequency

Capacitors drain their energy and any real-world capacitor may be thought of as an ideal capacitance linked in series to an equivalent series resistance (*ESR*). It is possible that the occurrence of *ESR* in a capacitor is caused by the dipole relaxation and electronic conduction in the dielectrics. The dissipation factor (*DF*) is used to indicate the purity of a capacitor and may be defined as the ratio of real power loss in the *ESR* to the reactive power fluctuating over the capacitive reactance (*XC*).

Mathematically,

(4) $$DF\;=\;\;\;\frac{ESR}{\left|Xc\right|}$$

For an AC supply with frequency *f*:

(5) $$\left|Xc\right|\;=\;\;\;\frac{1}{\left(2\pi fC_{m}\right)}$$

where, *C_m_* = the capacitance in a medium.

Thus,

(6) $$DF\;=\;ESR\left(2\pi fC_{m}\right)$$

By combining Equations (4) and (5), we can deduce:

(7) $$DF\;=\;\left(2\pi fESRC_{o}\right)ĸ$$

Equation (7) states that the *DF* varies as a function of the dielectric constant (ĸ) if any dielectric with specific *ESR* is exposed to an AC supply. As a result, the higher the dielectric constant (ĸ) for dielectrics, the greater the energy storage potential.

**Figure 3:**
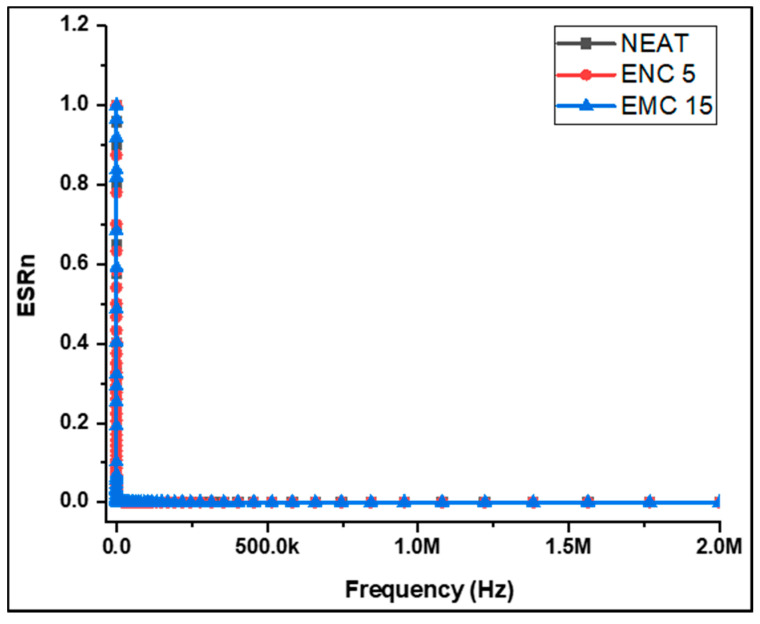
ESRn values of the samples against the frequency range.

Lower k and a lower *DF* are needed for insulation purposes. It is quite challenging to estimate the reading of the capacitor’s real resistance at lower and higher frequencies. This is due the fact that at low frequencies, impedance is high, and at high frequencies, the displacement current is high. The LCR meter is unable to calculate the exact phase angle at lower frequencies because the impedance reaches a particular threshold value and hence the *ESR* values at lower frequencies are unreliable. Conversely, a high displacement current at higher frequencies is due to the lower series resistance. As a result, resistance calculated at these frequencies may not be equal to the real resistance. For best measurement accuracy, the desired impedance in the current case was in the range of 100 to 100 k. Consequently, measurements were anticipated to be more reliable at higher frequencies, while low frequency results were ambiguous. For example, Figure 3 shows incorrect findings in the whole range of 10 Hz to 2 MHz and the term ESRn represents normalized values that can be calculated by dividing the specific *ESR* of each sample by the maximum *ESR*.

Below 20 kHz frequencies, an abrupt enhancement in normalized *ESR* varying with frequency was observed. This immediate rise can be associated with increases in impedance above the 100-kΩ limit. For these high impedances, the LCR meter cannot correctly calculate phase angles; therefore, both *ESR* and *Xc* should not be considered. In the case of the *DF* calculation, such impacts become even more prominent. As a result, the dielectric characteristics should be considered in a suitable frequency range in which the readings are reliable. Hence, for accurate measurements of *DF* and the dielectric constant ĸ, the test frequency range 60 kHz–2.0 MHz was selected.

### 3.2. Analysis in the Range of 60 kHz–2 MHz

**Figure 4:**
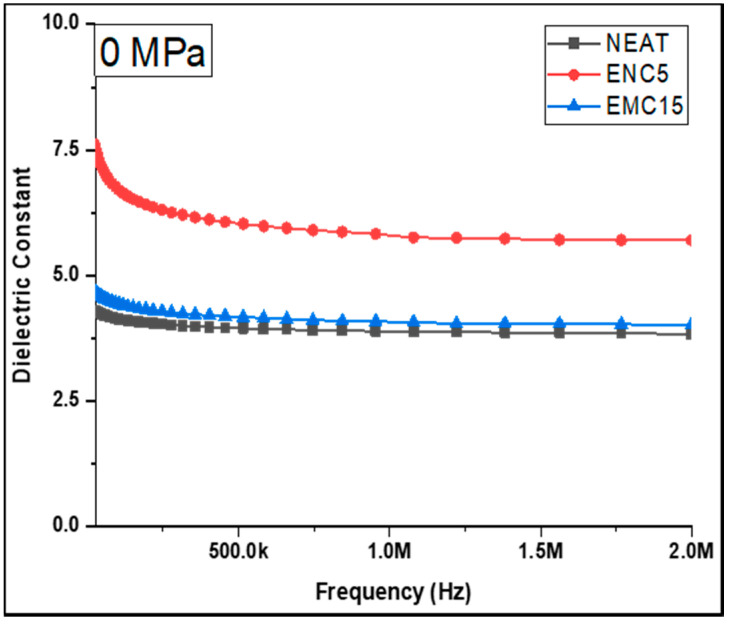
Dielectric constant of the uncompressed samples.

Dielectric constants and the dissipation factor were recorded for the epoxy and its composites. Figure 4 depicts the effects of the addition of silica filler in the epoxy on the dielectric constant (ĸ). The dependency of the frequency of the dielectric constant (ĸ) was studied. The neat epoxy showed the lowest k with an average value of 4.14 and EMC 15 showed an average value of 4.68. Among all samples, ENC 5 exhibited the highest response with an average value of 7.68. Generally, the value of the dielectric constant (ĸ) increased with the silica loading. In case of EMC 15, however, a decrement in the dielectric constant was shown with the maximum concentration. This might be due to the smaller surface area of micro-silica, which resulted in poor dispersion of the filler.

**Figure 5:**
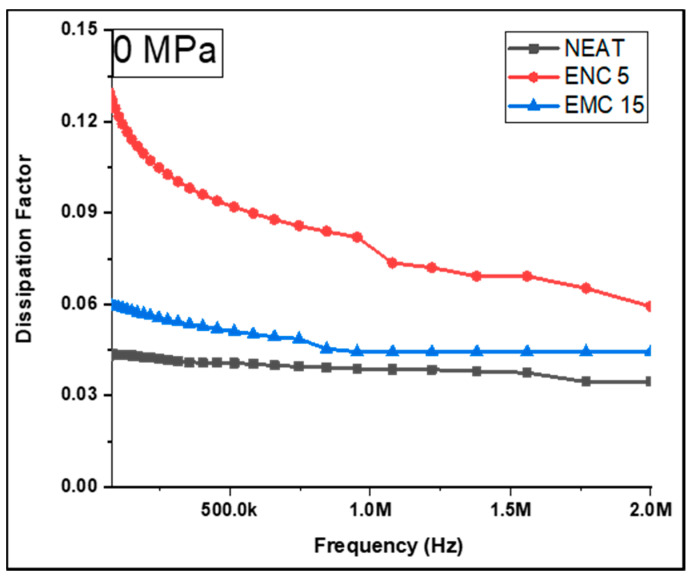
Dissipation factor of the uncompressed samples.

Based on the foregoing findings, it can be established that the dielectric constant of the epoxy matrix can improve with the inclusion of silica filler. In comparison to micro-silica, however, this effect was more evident in nano-silica. This property of the epoxy–SiO2 combination can be ascribed to the fine dispersion of nano-silica and the enhancement of the surface area. In terms of the dissipation factor, Figure 5 illustrates the purity of the capacitive nature of the neat epoxy and its composites, demonstrating that *DF* increased as the test frequency increased. The explanation for this concerns a large reduction in the displacement current at lower frequencies. Similarly, a decrease in the dielectric constant was observed in conjunction with an increase in *DF* as frequency increased. The neat epoxy showed the lowest dissipation factor with an average of 0.03 and EMC 15 with an average value of 0.05. Furthermore, ENC 5 had an average *DF* of 0.18.

It can be inferred from the above results that EMC 15 showed contradictory behavior due to restricted molecular level interactivity. Another cause of the contradictory response of EMC 15 could be the non-uniform distribution of the micro-filler and the filler–filler interactions, which leads to many voids with trapped air. Equation (4) is completely consistent with this response.

Due to the existence of an ample amount of silanol functional groups (Si-OH) at the surface of nano-silica, epoxy nanocomposites showed excellent dielectric responses contrary to the other samples. These silanol groups provided solidity to the overall epoxy matrix by providing strong binding with hydrogen. Hence, intactness of the overall epoxy composite system was achieved due to SiO_2_ nanoparticle and polymer interactivity at interaction regions [18,19]. In the case of the micro-composite (EMC 15), however, despite its 15% concentration, improvement in dielectric properties was not achieved because of the nine times or above silanol groups on the nanoparticles’ surface. This interprets the intent to keep low concentrations of nano-silica as compared to micro-silica to achieve improvements in the dielectric characteristics of the epoxy-silica composite system [20]. Furthermore, the concentration of nano-silica beyond 5% may create agglomeration and decrease the inter-filler distances due to these phenomenon-like significant properties of polymerics that may be compromised at higher nanofiller-loadings [21]. In a study carried out by M. Fairus [22], they investigated the dielectric strength of SiR/EPDM nanocomposites and reported that nanofiller concentrations above 5% caused agglomeration in the polymer matrix and enhanced mobile charge carriers, which eventually reduced the dielectric strength of nanocomposites. Conversely, the loading of micro-fillers can be enhanced above 40%, but increased filler-loadings may create significant impacts on inter-filler interactions which worsen dielectric properties and thermal stability [23,24]. Consequently, considering all the above constraints, an optimum loading of micro-fillers at about 10–20% can be used to restrain filler–filler interactivity [25,26].

### 3.3. Ramped Compression Effects

To explore the electromechanical characteristics of the epoxy–SiO_2_ system, the samples were compressed at multiple steps of pressures: 5 MPa, 10 MPa, 15 MPa, 20 MPa, and 25 MPa at 70 °C. After each compression step, the dielectric constant (ĸ) of all samples were evaluated and the comparative analysis of samples using graphical representation was investigated. Through all this experimental work, one trend can be clearly observed for all the working samples that dielectric constants irregularly enhanced by increasing compressive pressure. This trend, however, was more prominent in the case of micro-composites.

**Figure 6:**
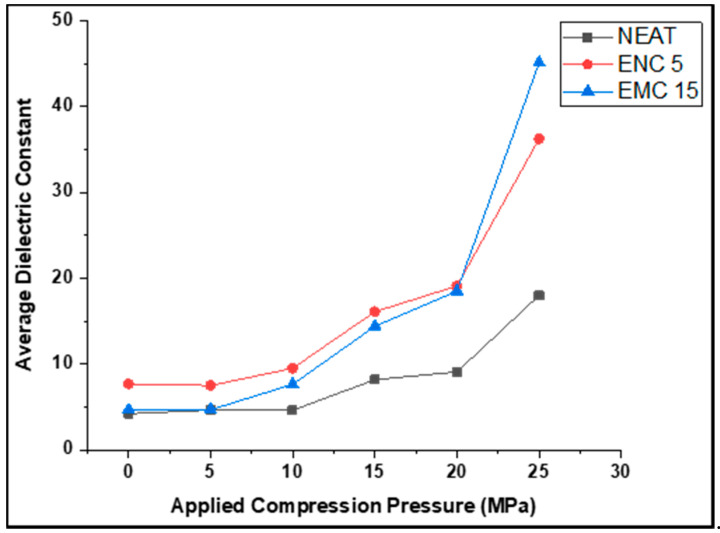
Average dielectric constant of samples.

**Figure 7:**
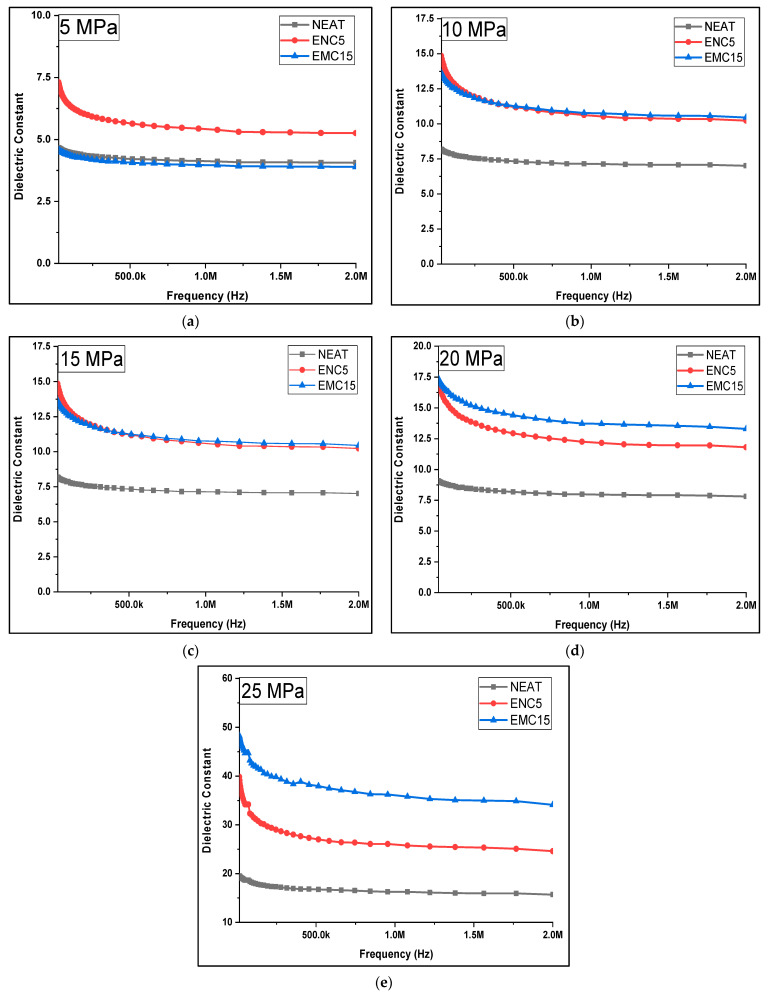
Dielectric constant of samples after compressions at (**a**) 5 MPa, (**b**) 10 MPa, (**c**)15 MPa, (**d**) 20 MPa, and (**e**) 25 MPa.

For comparison of all the samples, Figure 6 and Table 2 are presented, which show the average dielectric constants of samples against each compression step. In a similar manner, Figure 7 displays the trends of the dielectric constants of each sample at the chosen frequency range. For neat epoxy, at 5 and 10 MPa, the average dielectric constant remained almost the same, but at 15 MPa and 20 MPa, it improved 1.94 and 2.1 times, respectively, for the uncompressed sample. By applying 25 MPa, it increased 4.267 times for the uncompressed sample. Similarly, in the case of ENC 5, the dielectric constant at 10 MPa, 15 MPa, 20 MPa improved by 1.23, 2.09, and 2.487 times, respectively, as compared to the uncompressed sample, and at 25 MPa, the dielectric constant reached to 36.24, which is 4.718 times the uncompressed sample. The most irregular and prominent response could be seen in the case of EMC 15 as compared to other samples, where the average dielectric constant jumped to an average of 45.15 from 4.68 after successive mechanical pressures up to 25 MPa. The dielectric constant of the nanocomposite remained least affected by compression, contrary to the neat and micro-composite. This is because of the interaction of nano-silica with the polymer chains, which restricted the motion of the polymer chain, and the electrical polarization was not aided by the polymer chains [23,27,28]. In the case of the micro-composite, the relatively larger size of micro-silica did not achieve as good filler dispersion as compared to the nanocomposite, which left voids in the composite that began to deplete, and interfacial polarization enhanced by increasing mechanical pressures.

**Figure 8:**
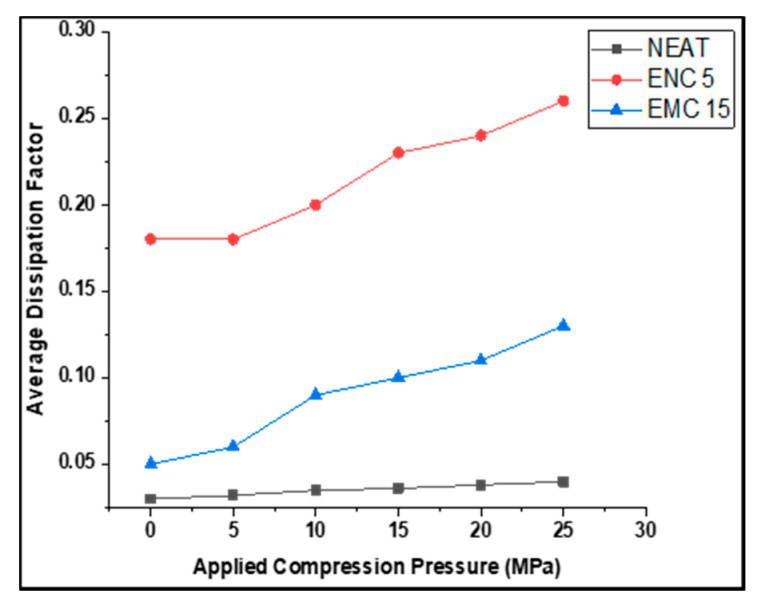
Average dissipation factor of samples.

**Figure 9:**
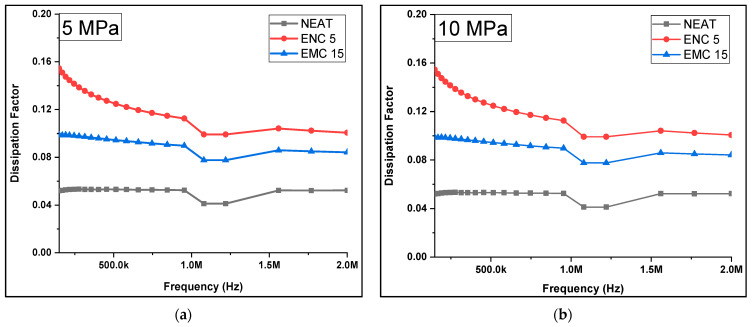

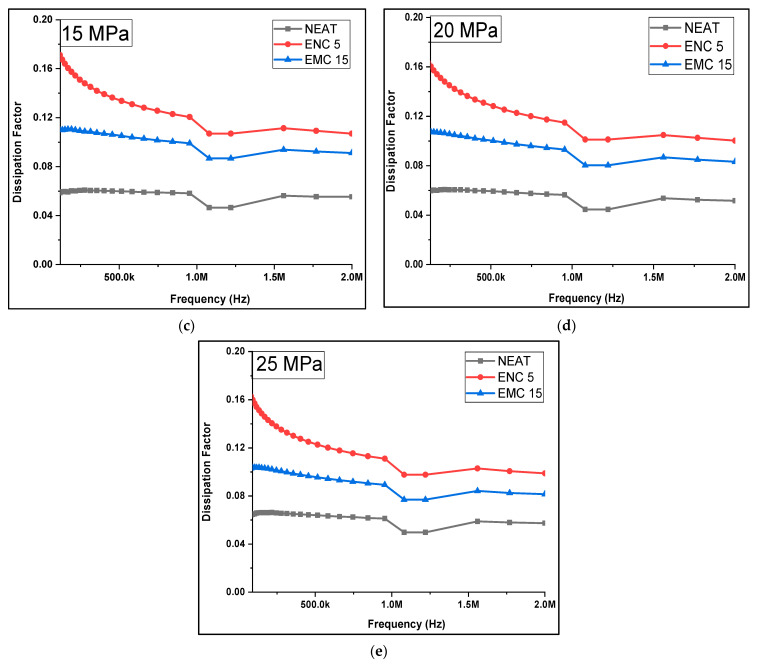
Dissipation factor of samples after compressions at (**a**) 5 MPa, (**b**) 10 MPa, (**c**) 15 MPa, (**d**) 20 MPa, (**e**) 25 MPa.

The dissipation factor was recorded against the ramped compression. Figure 8 and Table 3 are given to demonstrate the calculated average dissipation factors of all the samples at the selected frequency range against the corresponding compression pressure, while Figure 9 shows the trends of the dissipation factor against the frequency range. It can be observed that almost a similar pattern was followed by dielectric constant (ĸ). There was less change in *DF* in the neat epoxy after each compression step. At 5 MPa and 10 MPa, *DF* remained same, but at 15 MPa, 20 MPa, and 25 MPa, it was increased to 0.04 from 0.03 as compared to the uncompressed. In the case of ENC 5, *DF* increased to 0.25 from 0.18 at 10 MPa and remained almost the same up to 25 MPa. In comparison to the uncompressed sample, a 1.33-times increase was recorded. In the case of EMC 15, *DF* was most affected by increasing mechanical compression and increased 2.2 times from 0.04 to 0.11. This could be attributed to the inhomogeneities of micro-fillers. The dissipation factor (dielectric loss) was influenced by the enhancement of interfacial polarization due to the presence of mobile ionic impurities [27,28].

Similar outcomes were also reported and prior literature on other classes of uncompressed epoxy composites such as in Singha et al. [23] stated high permittivity and tan delta values for Epoxy-TiO2/Zinc Oxide micro and nanocomposites, and concluded that interfacial properties are pivotal factor for the dielectric behavior of nanocomposites. Similarly, Yu, J. [29] obtained enhanced breakdown strength and dielectric constants for epoxy-alumina nanocomposites.

## 4. Conclusions

The dielectric characteristics of epoxy composites with nano and micro-silica fillers were investigated under mechanical pressures from 0 MPa to 25 MPa with ramped compression steps of 5 MPa at 70 °C. Before applying compressions, nanocomposites with 5% nano-silica (ENC 5) exhibited a maximum dielectric constant with an average of 7.68 and *DF* with an average of 0.18. After each compression step, the dielectric constant (ĸ) and dissipation factor (*DF*) of neat epoxy, epoxy-nanocomposite (ENC 5), and epoxy-micrcomposite (EMC 15) were measured and a comparative analysis was conducted. Major variations in results were experienced above 15 MPa. The dielectric constant and *DF* of ENC 5 increased 2.48 and 1.33 times at 20 MPa. Similarly, at 25 MPa, an increase of 4.71 and 1.44 times was recorded. In the case of EMC 15, at 20 MPa and 25 MPa, the dielectric constant and *DF* were enhanced by 3.97 and 2 times, and by 9.63 and 2.2 times, respectively. It was clearly observed that microcomposites with 15% filler-loading were badly influenced by compressions as compared to nanocomposites.

## Figures and Tables

**Table 1:** Synthesized epoxy composites.

Sample	Code
Neat Epoxy	Neat
Epoxy—with 5 wt.% Nano-silica Filler	ENC 5
Epoxy—with 15 wt.% Micro-silica Filler	EMC 15

**Table 2:** Average dielectric constant of samples at different pressures.

Samples	0 MPa	5 MPa	10 MPa	15 MPa	20 MPa	25 MPa
NEAT	4.22	4.63	4.61	8.18	9.08	18.01
ENC 5	7.68	7.50	9.51	16.10	19.10	36.24
EMC 15	4.68	4.67	7.65	14.41	18.52	45.14

**Table 3:** Average dissipation factor of samples at different pressures.

Samples	0 MPa	5 MPa	10 MPa	15 MPa	20 MPa	25 MPa
NEAT	0.03	0.03	0.03	0.04	0.04	0.04
EMC 15	0.05	0.06	0.09	0.10	0.10	0.11
ENC 5	0.18	0.18	0.24	0.25	0.24	0.26

## Data Availability

Authors confirm the availability of all the supporting material and findings in the manuscript.
